# Development of a nomogram based on radiomics and semantic features for predicting chromosome 7 gain/chromosome 10 loss in IDH wild-type histologically low-grade gliomas

**DOI:** 10.3389/fonc.2023.1196614

**Published:** 2023-09-15

**Authors:** Xin Kong, Yu Mao, Fengjun Xi, Yan Li, Yuqi Luo, Jun Ma

**Affiliations:** ^1^ Department of Radiology, Beijing Tiantan Hospital, Capital Medical University, Beijing, China; ^2^ Department of Radiology, Beijing Fengtai Hospital, Beijing, China

**Keywords:** machine learning, radiomics, glioma, nomogram, linear discriminant analysis

## Abstract

**Purpose:**

To predict chromosome 7 gain and chromosome 10 loss (+7/-10) in IDH wild-type (IDH-wt) histologically low-grade gliomas (LGG) by machine learning models based on MRI radiomics and semantic features.

**Methods:**

A total of 122 patients diagnosed as IDH-wt histologically LGG were retrospectively included in this study. The patients were randomly divided into a training group and a test group in a ratio of 7:3. The radiomics features were extracted from axial T1WI, T2WI, FLAIR and CET1 sequences, respectively. The distance correlation (DC) and least absolute shrinkage and selection operator (LASSO) were used to select the radiomics signatures. Three machine learning algorithms including neural network (NN), support vector machine (SVM), and linear discriminant analysis (LDA) were used to construct radiomics models. In addition, a nomogram was developed by combining the optimal radiomics signature with clinical risk factors, and the potential clinical utility of the nomogram was evaluated using decision curve analysis.

**Results:**

The LDA+DC model was identified as the optimal classifier among the six radiomics models. Necrosis was determined as a risk factor for +7/-10 in IDH-wt histologically LGG. The nomogram achieved the best performance, with an AUC of 0.854 and an accuracy of 0.778 in the independent test group. The decision curve of the nomogram confirmed its clinical usefulness in a wide range of thresholds.

**Conclusion:**

The nomogram combining radiomics and semantic features can predict the +7/-10 status effectively, which may contribute to the risk stratification and individualized treatment planning of patients with IDH-wt histologically LGG.

## Introduction

Gliomas are the most common primary tumors of the central nervous system and are classified by the World Health Organization (WHO) as low-grade gliomas (LGG, grade 2-3) and glioblastomas (GBM, grade 4) based on the histological findings. The LGG is characterized by a widespread malignant potential and primarily affects young people, with a median overall survival of approximately 7 years (1). GBM is extremely malignant and mainly affects the elderly, with a median survival of only 8 months after diagnosis (2, 3). Gliomas have a unique genomic profile, and molecular markers have been identified as an essential basis for glioma classification in the 2021 WHO Classification of Central Nervous System Tumors. IDH wild type (IDH-wt) histologically LGG with chromosome 7 gain and chromosome 10 loss (+7/-10) in adults have been reclassified as GBM due to their clinical outcome and prognosis being similar to that of GBM (4, 5). In addition, these tumor entities will also be managed in the same manner as GBM.

Maximal safe resection plays a vital role in the management of gliomas and is clearly the preferred choice for improving survival. However, LGG and GBM have different treatment approaches after surgery. GBM tends to benefit from postoperative radiation therapy and chemotherapy due to its highly aggressive nature, whereas the choice of postoperative treatment for LGG primarily depends on molecular subgroups. High-risk LGG patients harboring IDH mutations can benefit from adjuvant PCV (procarbazine, lomustine (CCNU), and vincristine) after radiotherapy, leading to a significant extension in overall survival (OS) and progression-free survival (PFS). IDH wild-type LGG patients cannot benefit from adjuvant PCV after radiotherapy, and may require more aggressive treatment approaches (6–8). Furthermore, the expression status of molecular characteristics guides the choice of treatment options. The loss of Chr10 is an important pathway that increases tumor cell sensitivity to alkylating agents. Therefore, adjuvant chemotherapy with temozolomide or nitrosourea is the optimal choice for patients with Chr10 loss (3). In addition, the gain of Chr7 was associated with a 4.7-fold increased risk of tumor recurrence, while the loss of Chr10 was linked to a shorter survival (9). Therefore, identifying this pair of major oncogenic-driven genes in IDH-wt histologically LGG not only helps to diagnose the molecular glioblastoma but also facilitates the regulation of the oncogenic signaling pathway at the molecular level for individualized treatment and prognosis prediction (10).

Stereotactic biopsy is the standard diagnostic procedure for the diagnosis of brain tumors. However, it is an invasive examination and is severely limited by spatial heterogeneity of tumor tissue and inter-observer variability (3). In addition, genetic testing is costly and not available in all basic healthcare units. Therefore, it is imperative to develop a rapid, non-invasive, and reliable method to predict the status of +7/-10 in IDH-wt histologically LGG. Radiomics can transform conventional imaging data of lesions into high-resolution, high-throughput information for quantitative analysis to characterize tumor heterogeneity (11, 12). Since the concept of radiomics was put forward, it has been widely utilized in the differential diagnosis, gene expression status and prognosis prediction of glioma (13–15). Meanwhile, clinical and radiological semantic features have also been demonstrated to effectively reveal the biological phenotype of glioma (16, 17). The purpose of this study was to predict the status of +7/-10 in IDH-wt histologically LGG patients by machine learning models based on radiomics and semantic features to assist clinicians in accurate diagnosis, prognosis-based stratification and individualized treatment for glioma patients.

## Materials and methods

### Participants

**Figure 1:**
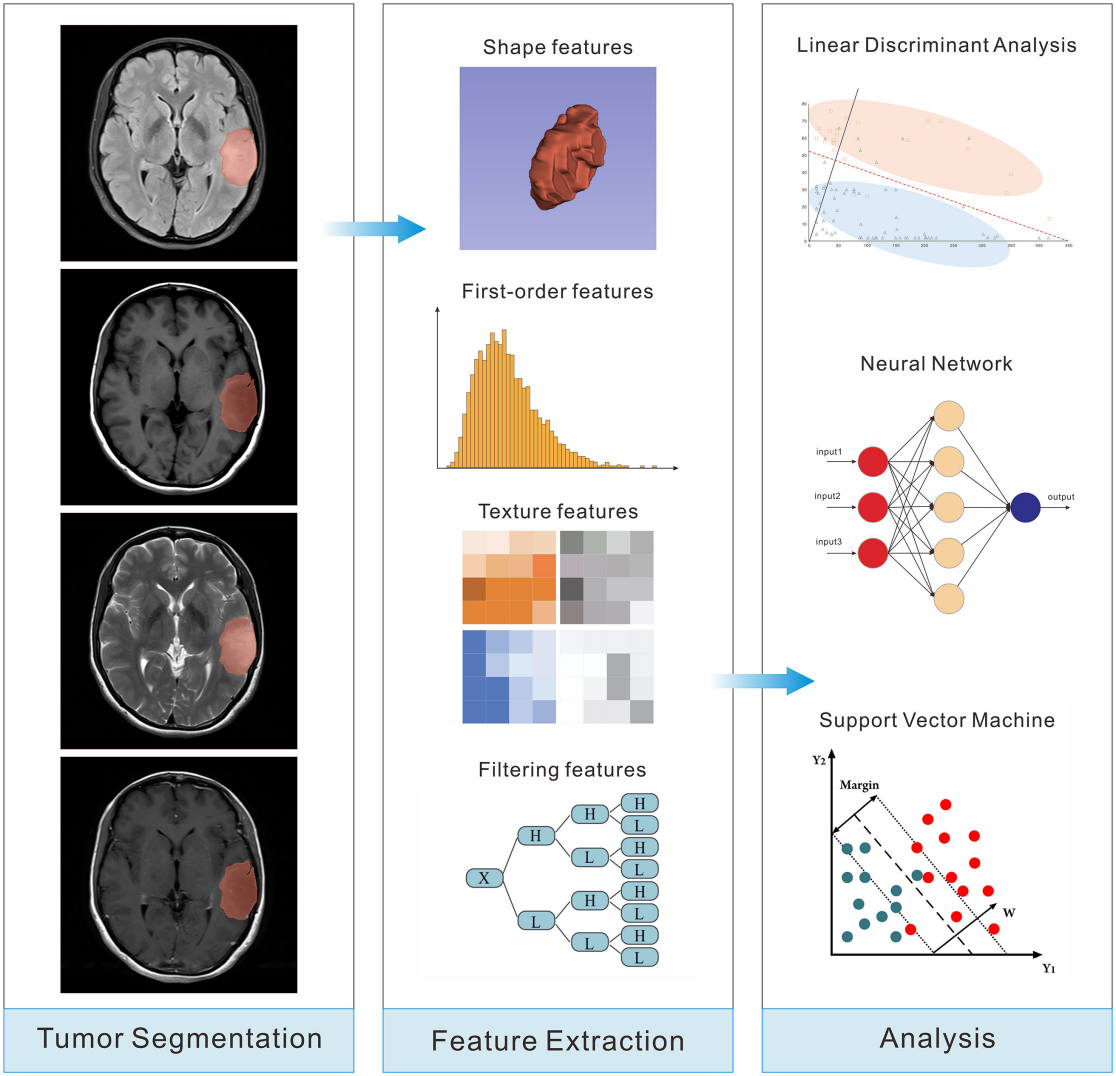
The flowchart of this study.

This study was approved by the institutional review board of our institute with the informed consent waived (IRB: KY2022-214-03). We retrospectively enrolled glioma patients who underwent surgery at our institution from January 2020 to June 2022. The inclusion criteria were (1): patients with first surgery and a histological diagnosis of LGG (2); expression status of Chr7 and Chr10 was determined and IDH was a wild type (3); complete axial T1WI, T2WI, T2-FLAIR and CET1 sequences were available in preoperative MRI examination (4); images were acquired using a 3.0T scanner. The exclusion criteria were (1): patients who had received glioma-related treatment such as radiotherapy and chemotherapy before MRI examination (2); MRI images that did not meet software processing requirements (3); juvenile patients. All the patients were randomly divided into a training group and a test group in a ratio of 7:3. The flow chart of the study was shown in **Figure 1**.

### Image acquisition and pre-processing

All MR images were acquired using 3T magnetic resonance scanners, including Magnetom Trio Tim (Siemens), Magnetom Verio (Siemens), Magnetom Prisma (Siemens) and Discovery 750 (GE Medical Systems). The acquisition protocol was shown in **Supplementary S1**. In this study, two pre-processing methods in 3Dslicer software (https://www.slicer.org/) were employed to eliminate potential effects caused by the discrepancies in different scanners and acquisition parameters. Firstly, bias field correction was performed on all images using the N4ITK toolkit in 3Dslicer to eliminate the image noise caused by the inhomogeneity and fluctuation of the scanning magnetic field in different scanners and within the same scanner (18, 19). N4ITK is a non-parametric non-uniform intensity normalization algorithm that uses a b-spline approximation to achieve the best fit and can also compensate for the lack of standard units in MR images to some extent. Subsequently, all images were resampled to 1×1×1mm by tri-linear interpolation to correct for the impact caused by different acquisition parameters (20, 21).

### Segmentation and feature extraction

The segmentation process was performed by a neuro-radiologist with more than 5 years of experience using 3Dslicer software, and the neuro-radiologist was blind to the pathological results before segmentation. According to the Brain Tumor Image Segmentation Benchmark (BRATS), the region of interest (ROI) was delineated as the entire tumor area, including edema, enhancing core, non-enhancing core, and necrotic/cystic core (22). First, the three-dimensional ROI was manually delineated by the neuro-radiologist layer by layer on the FLAIR sequence. Then the ROI was registered to the T1WI, T2WI and CET1 sequences and the ROI profile was manually adjusted by the neuro-radiologist. The Pyradiomics library (http://pyradiomics.readthedocs.io/) was applied to extract radiomics features from the four sequences. A total of 6380 features including shape, first-order, texture and filtering were extracted from the four sequences. A detailed introduction of the features was provided in **Supplementary S2**. In addition, 30 cases were randomly selected and the segmentation process was repeated by another neuro-radiologist with more than 10 years of experience. The interclass correlation coefficient (ICC) between the two radiologists was calculated to evaluate the accuracy of segmentation and the reproducibility of radiomics features.

### Radiomics feature selection

The feature selection and modeling were conducted in the training group. Since many radiomics features have different dimensions and cannot be directly compared with each other, all features were normalized by Z-score before feature selection to eliminate the different dimensionality. Subsequently, we employed 3 steps for the selection of radiomics features. Firstly, univariate analysis was conducted based on variable distribution to eliminate redundant features and reduce the computational burden of the model. Secondly, we calculated the Spearman correlation coefficients between each pair of features to avoid severe feature collinearity. In a pair of features with a correlation coefficient greater than 0.9, the one with a lower weight was eliminated. Finally, the optimal feature signatures were selected by 2 common feature selection algorithms including distance correlation (DC) and least absolute shrinkage and selection operator (LASSO), respectively.

### Development of the radiomics models

Three machine learning algorithms including neural network (NN), support vector machine (SVM), and linear discriminant analysis (LDA) were used to develop the radiomics models, respectively. Each model underwent 10-fold cross-validation in the training group to tune the parameters and obtain the true classification accuracy. The independent test group was used to further evaluate the performance of the models. The AUC value, sensitivity, specificity, and accuracy of each model were calculated in the training and test groups, respectively. The model with the highest AUC value in the test group was considered the best radiomics model, and its signature was also defined as the optimal radiomics signature.

### Development of the clinical model and nomogram

Based on previous studies and the VASARI features of glioma, we selected 12 semantic features including age, gender, location, side, margin, contrast enhancement, necrosis, multifocal, restricted diffusion, deep white matter invasion, hemorrhage, and ependymal involvement as potential clinical risk characteristics for +7/-10 in IDH-wt histologically LGG (23, 24). The detailed definitions of semantic features were provided in **Supplementary S3**. The conventional radiological features were evaluated by two neuro-radiologists with more than 5 years of experience in a blinded manner to clinical and pathological information. Any disagreements between the two would be reassessed by a senior neuro-radiologist with more than 10 years of experience. Univariate analysis was used to identify the risk factors for +7/-10. Then, a clinical model for predicting +7/-10 was established using logistic regression in the training group and validated in the test group.

To provide a reliable and convenient tool for clinicians to predict +7/-10 in IDH-wt histologically LGG, a nomogram incorporating radiomics signature and clinical risk factors was developed in the training group and validated in the test group. The C-index of the nomogram was calculated and the calibration curve was plotted to evaluate the consistency between the predicted probability and the true results as well as the stability of the nomogram. In addition, decision curve analysis was used to measure the benefits at different prediction thresholds to evaluate the clinical applicability of the nomogram.

### Statistics

All the statistical analysis in our study were performed by Rstudio (version: 1.2.1335; https://www.rstudio.com/). For univariate analysis, a t-test or Mann-Whitney U-test was performed according to the distribution of measurement data. Chi-square test or Fisher’s exact probability test was applied to count data. The “caret” package was used for modeling, and the “pROC” package was used to plot receiver operating characteristic (ROC) curves and calculate the AUC value, sensitivity, specificity, and accuracy. *P <*0.05 was considered as significantly different.

## Results

### Patient characteristics

A total number of 122 eligible patients were included in this study. Among them, there were 42 patients with +7/-10 and 80 patients without +7/-10. The mean age of patients with +7/-10 was 47.90 ± 12.46, with 18 males and 24 females, while the mean age of patients without +7/-10 was 44.20 ± 14.56, with 38 males and 42 females. All the patients were randomly divided into a training group (n=86) and a test group (n=36) in a ratio of 7:3. The proportion of patients and semantic features did not differ significantly between the training and test groups (**Table 1**), suggesting that this grouping was justified.

**Table 1:** The baseline data in the training and test groups.

Characteristic	Training group (n=86)	Test group (n=36)	*P*-value
Age (year)	45.55 ± 13.53	45.31 ± 15.03	0.934
Gender			0.324
Male	37	19	
Female	49	17	
Proportion of patients			>0.999
With +7/-10	30	12	
Without +7/-10	56	24	
Location			0.154
Frontal lobe	38	21	
Others	48	15	
Side			0.998
Right	34	14	
Both	7	3	
Left	45	19	
Margin			0.291
Distinct	46	23	
Indistinct	40	13	
Contrast enhancement			0.528
Yes	40	19	
No	46	17	
Necrosis			0.560
Yes	31	11	
No	55	25	
Multifocal			0.830
Yes	13	6	
No	73	30	
Restricted diffusion			0.075
Yes	22	4	
No	64	32	
Deep WM invasion			0.691
Yes	44	17	
No	42	19	
Hemorrhage			>0.999
Yes	9	4	
No	77	32	
Ependymal involvement			0.964
Yes	41	17	
No	45	19	

WM, white matter.

### Feature selection

**Figure 2:**
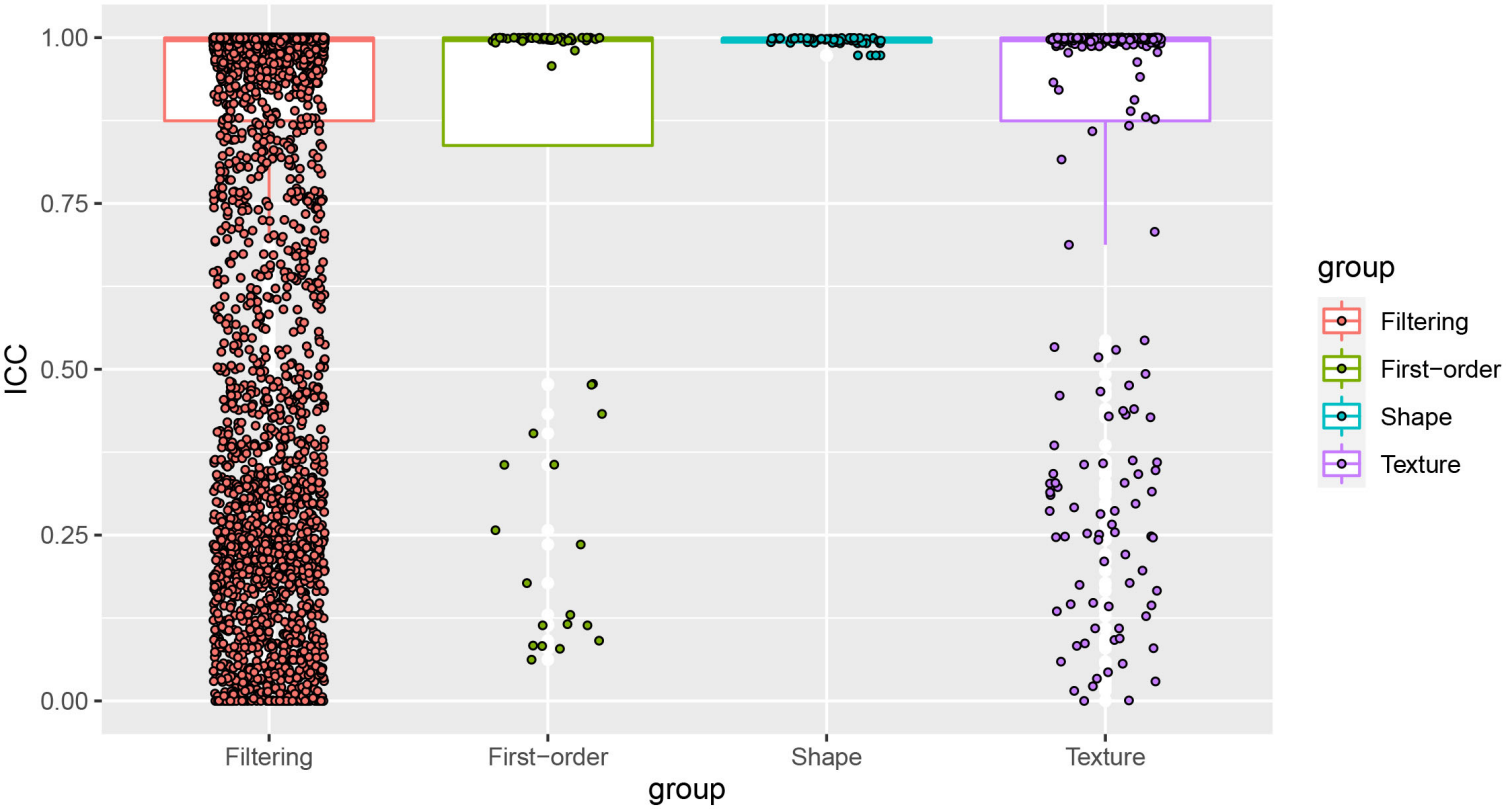
The ICC values of the 4 groups of features. Most of the features showed high reproducibility, and the features with ICC values less than 0.75 would be eliminated.

The average ICC value of the 6,380 pairs of features was 0.821, and the median ICC value was 0.997, indicating a high consistency. The boxplot of the ICC between the features extracted by two neuro-radiologists was shown in **Figure 2**. Since there are numerous mathematically transformed filtering features in our study, it may be too idealistic to require a high degree of consistency for all features. Therefore, We took 0.75 as the threshold for qualifying reproducibility and retained 4920 robust features with ICC greater than 0.75. Subsequently, 35 radiomics features were retained by univariate factor analysis and Spearman correlation analysis. Finally, LASSO selected 3 features based on the 1-SE criterion with a λ value of 0.1039153, while DC selected the top 10 most important features based on the distance correlation coefficient. The detailed information of the selected features was provided in **Supplementary S4**.

### Development and validation of radiomics models

**Figure 3:**
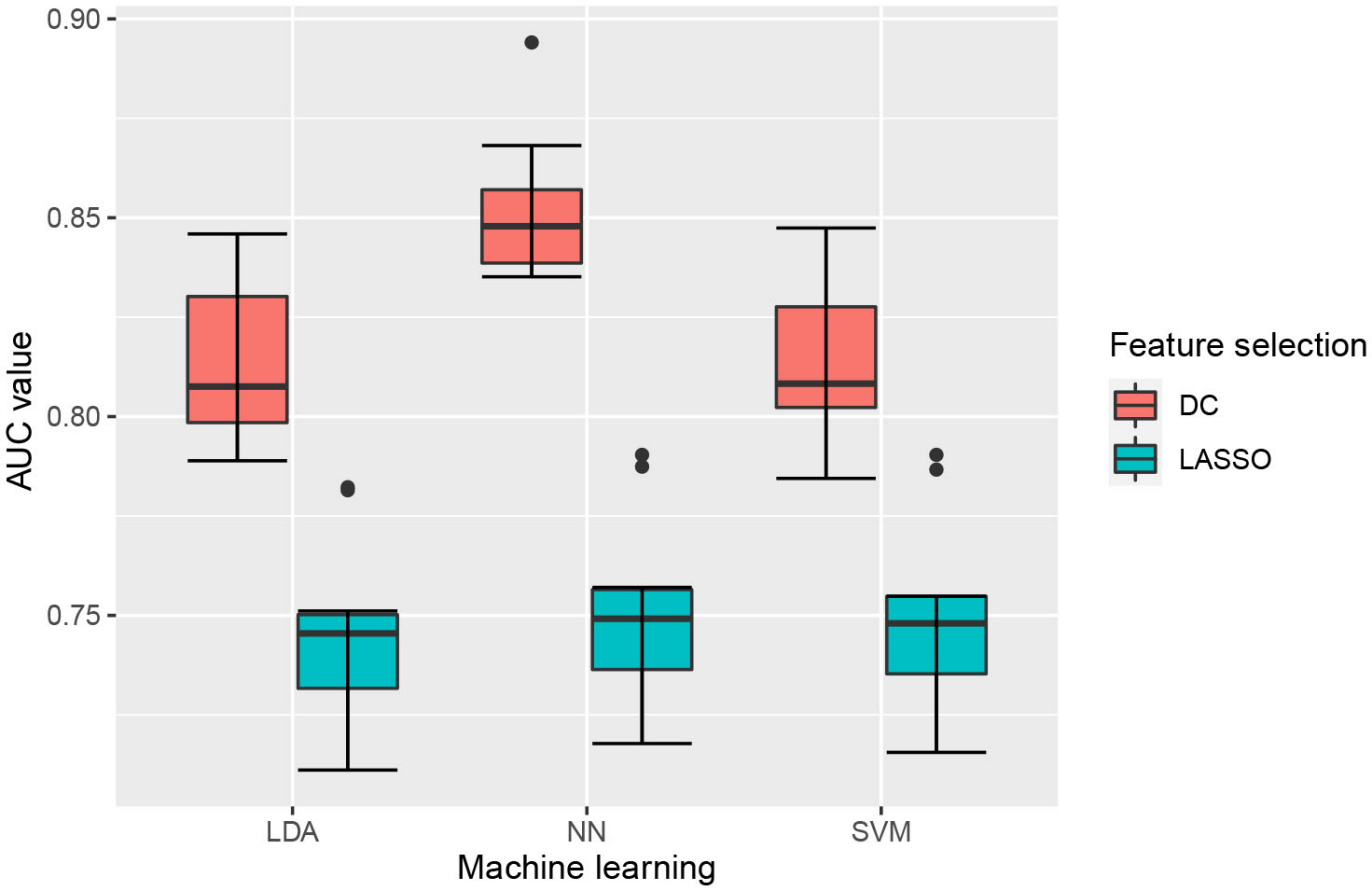
Boxplot of AUC values obtained by 10-fold cross-validation in the training group. Each model had 10 AUC values, and the median AUC values of models based on DC signature were superior to that based on LASSO signature.

**Figure 4:**
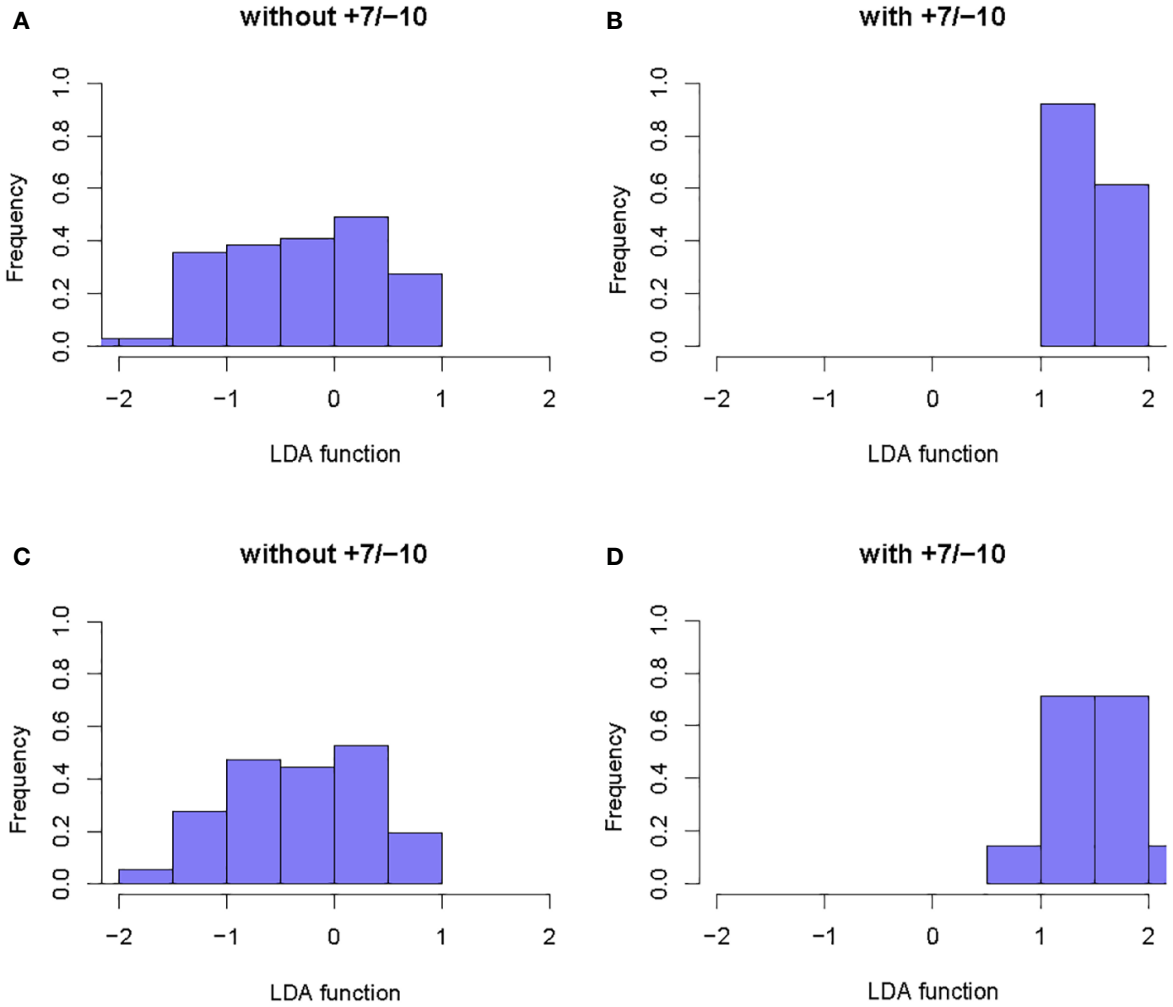
Example of the performance of the LDA-based models in determining the distribution of functions for patients with and without +7/-10. **(A, B)**: Function distribution of the LDA+DC model; **(C, D)**: Function distribution of the LDA+LASSO model. The observed minimal overlaps between the two groups indicated that the models based on LDA algorithm had a high discriminative ability.

**Figure 5:**
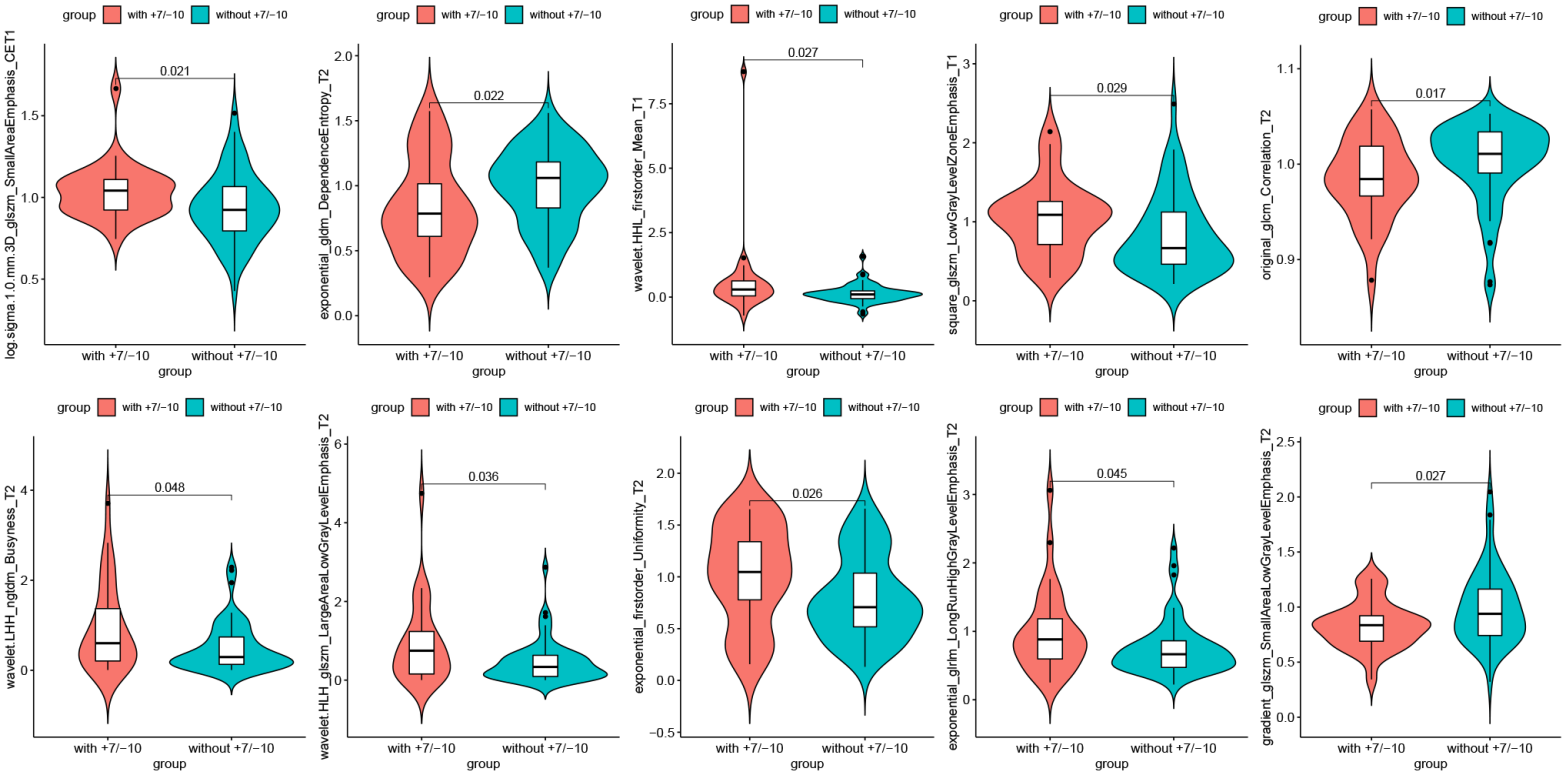
Radiomics features selected by the DC algorithm. The 10 features were statistically significantly different between the patients with and without +7/-10.

A total of 6 radiomics models were established combining 2 feature selection methods and 3 machine learning algorithms. The performances of these six models in the training and test groups were shown in **Table 2**. The AUC values of these models obtained from 10-fold cross-validation in the training group was shown in **Figure 3**. The median AUC values of the models based on DC signature were all higher than that of the models based on LASSO signature. Moreover, the LDA + DC model was defined as the optimal radiomics model due to its best performance in the independent test group, with an AUC of 0.736 and an accuracy of 0.750. Therefore, the feature set selected by the DC algorithm was defined as the optimal radiomics signature. **Figure 4** illustrated the performance of the LDA-based models in terms of the canonical function distribution over the training epoch. The 10 radiomics features selected by the DC algorithm were shown in **Figure 5**. One of them was an original feature (original_glcm_Correlation_T2), and the others were all filtering features. All radiomics features differed significantly between the IDH-wt histologically LGG patients with and without +7/-10 (*P <*0.05).

**Table 2:** The performance of the radiomics models.

Models	Group	AUC	Accuracy	Sensitivity	Specificity
LDA+DC	Training group	0.813	0.698	0.333	0.893
	Test group	0.736	0.750	0.500	0.875
LDA+LASSO	Training group	0.745	0.733	0.367	0.929
	Test group	0.597	0.694	0.250	0.917
SVM+DC	Training group	0.814	0.686	0.100	1.000
	Test group	0.726	0.639	0.083	0.917
SVM+LASSO	Training group	0.749	0.663	0.133	0.946
	Test group	0.608	0.750	0.250	1.000
NN+DC	Training group	0.852	0.861	0.900	0.839
	Test group	0.602	0.611	0.667	0.583
NN+LASSO	Training group	0.751	0.756	0.533	0.875
	Test group	0.608	0.639	0.417	0.750

### Development and validation of the clinical model and nomogram

**Figure 6:**
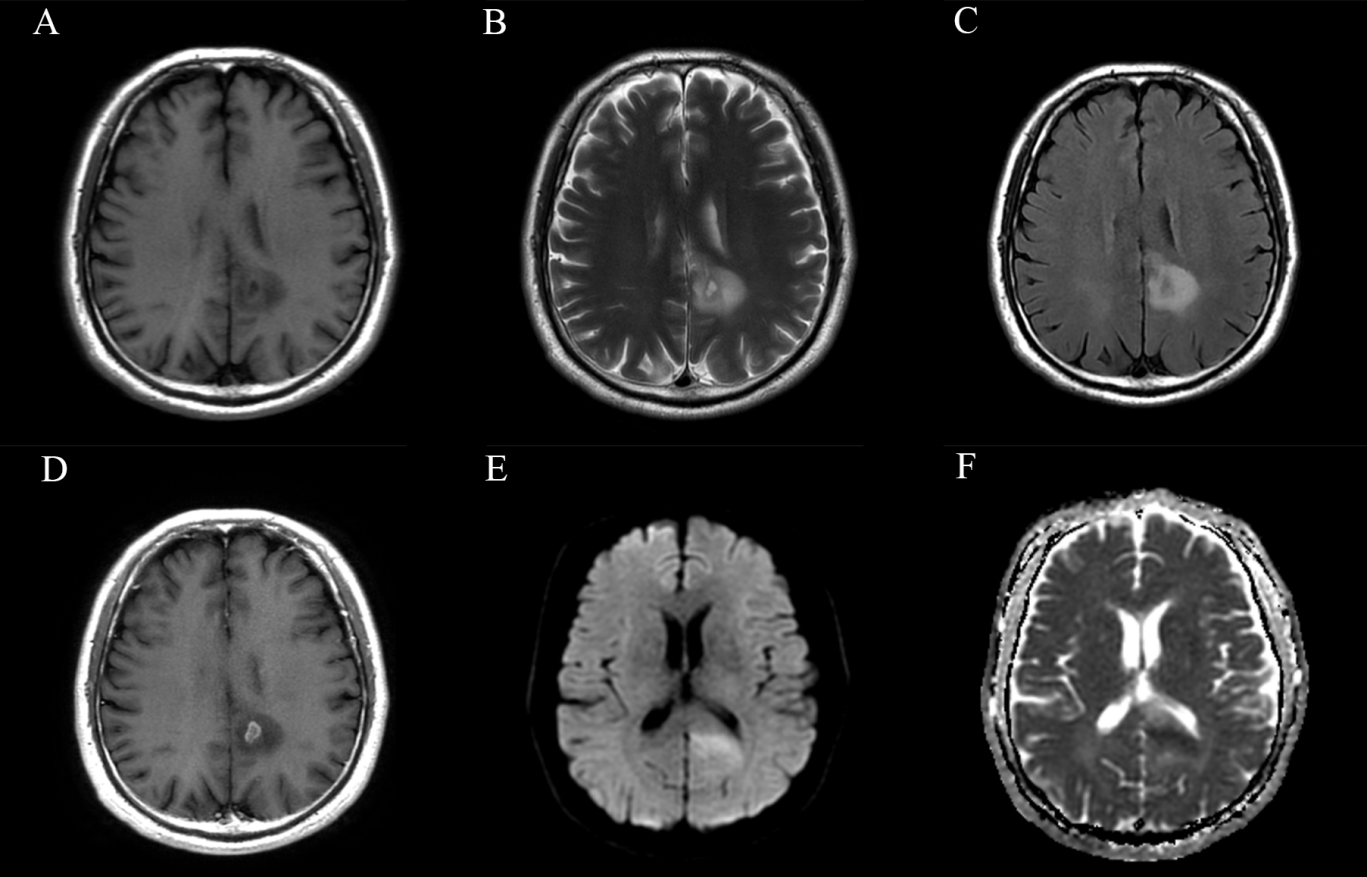
Magnetic resonance images of a representative case with +7/-10. **(A)** T1-weighted, **(B)** T2-weighted, **(C)** FLAIR, **(D)** CET1, **(E)** DWI and **(F)** ADC images of a 58-year-old male patient with a histopathological diagnosis of grade 3 astrocytoma. The lesion involves the left parietal lobe and corpus callosum. Small areas of necrosis and contrast-enhancement are observed in the central part of the lesion, and the lesion exhibits localized restricted diffusion. The molecular characteristics of this patient are as follows: IDH (–), EGFR (+), TERT promoter mutation, and amplification of chromosome 7/loss of chromosome 10. According to the 2021 CNS tumor classification guidelines, this case is now reclassified as molecular glioblastoma, indicating a relatively poor prognosis and clinical outcome.

**Figure 7:**
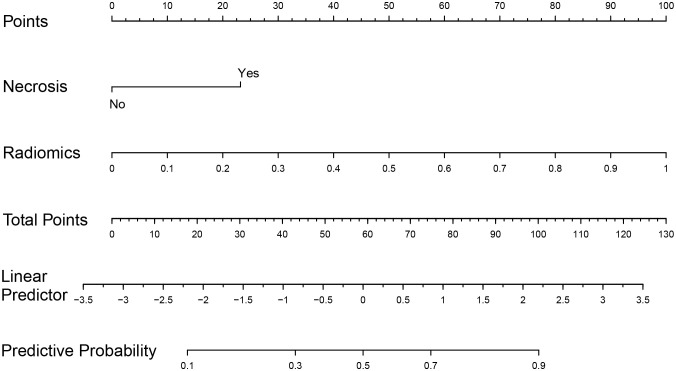
Nomogram based on multivariate logistic regression coefficients. Based on the summation of necrosis and radiomics scores, the probability of +7/-10 for IDH-wt LGG can be inferred.

**Figure 8:**
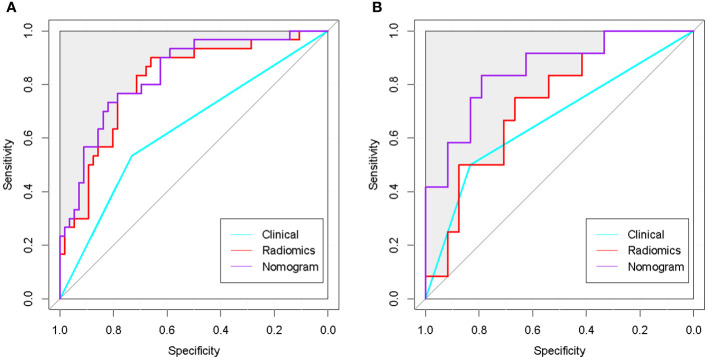
ROC curves of the optimal radiomics model, clinical model and nomogram. **(A)** ROC curves of the 3 classifiers in the training group. **(B)**: ROC curves of the 3 classifiers in the test group. The nomogram achieved the best performance in both the training and test groups.

Among all the included semantic features, there was a significant difference in necrosis between the IDH-wt histologically LGG patients with and without +7/-10 (**Table 3**). The incidence of necrosis in patients with +7/-10 was significantly higher than that in patients without +7/-10 in both the training group (*P*=0.015) and the test group (*P*=0.030). A representative case with +7/-10 was shown in **Figure 6**. Therefore, a clinical model was established using necrosis as a predictor. A nomogram was developed by combining the optimal radiomics signature and necrosis (**Figure 7**). The performance of the LDA+DC model, clinical model, and nomogram were shown in **Table 4** and the ROC curves of them were shown in **Figure 8**. The Delong test showed that the nomogram achieved the best predictive performance and significantly outperformed the clinical model in both the training (*P*<0.001) and test (*P*=0.039) groups.

**Table 3:** The baseline data in the training and test groups.

Characteristic	Training group (n=86)		*P*-value	Test group (n=36)		*P*-value
	With +7/-10 (n=30)	Without +7/-10 (n=56)		With +7/-10 (n=12)	Without +7/-10 (n=24)	
Age (year)	47.30 ± 11.83	44.61 ± 14.37	0.355	49.42 ± 14.34	43.25 ± 15.24	0.246
Gender			0.679			>0.999
Male	12	25		6	13	
Female	18	31		6	11	
Side			0.580			0.376
Right	10	24		5	9	
Both	2	5		2	1	
Left	18	27		5	14	
Location			0.567			0.282
Frontal lobe	12	26		5	16	
Others	18	30		7	8	
Margin			0.180			0.391
Distinct	19	27		6	17	
Indistinct	11	29		6	7	
Contrast enhancement			0.983			>0.999
Yes	14	26		6	13	
No	16	30		6	11	
Necrosis			0.015			0.030
Yes	16	15		7	4	
No	14	41		5	20	
Multifocal			0.769			0.635
Yes	5	8		1	5	
No	25	48		11	19	
Restricted diffusion			0.866			0.190
Yes	8	14		3	1	
No	22	42		9	23	
Deep WM invasion			0.768			>0.999
Yes	16	28		6	11	
No	14	28		6	13	
Hemorrhage			0.790			0.349
Yes	4	5		0	4	
No	26	51		12	20	
Ependymal involvement			0.891			0.194
Yes	14	27		8	9	
No	16	29		4	15	

WM, white matter.

**Table 4:** Comparison of the performance of the classifiers.

Models	Group	AUC	Accuracy	Sensitivity	Specificity	*p*-value (*vs*. Clinical)
Nomogram	Training group	0.838	0.767	0.567	0.875	<0.001
	Test group	0.854	0.778	0.583	0.875	0.039
Optimal radiomics (LDA+DC)	Training group	0.813	0.698	0.333	0.893	0.015
	Test group	0.736	0.750	0.500	0.875	0.818
Clinical	Training group	0.633	0.663	0.533	0.732	–
	Test group	0.667	0.722	0.500	0.833	–

**Figure 9:**
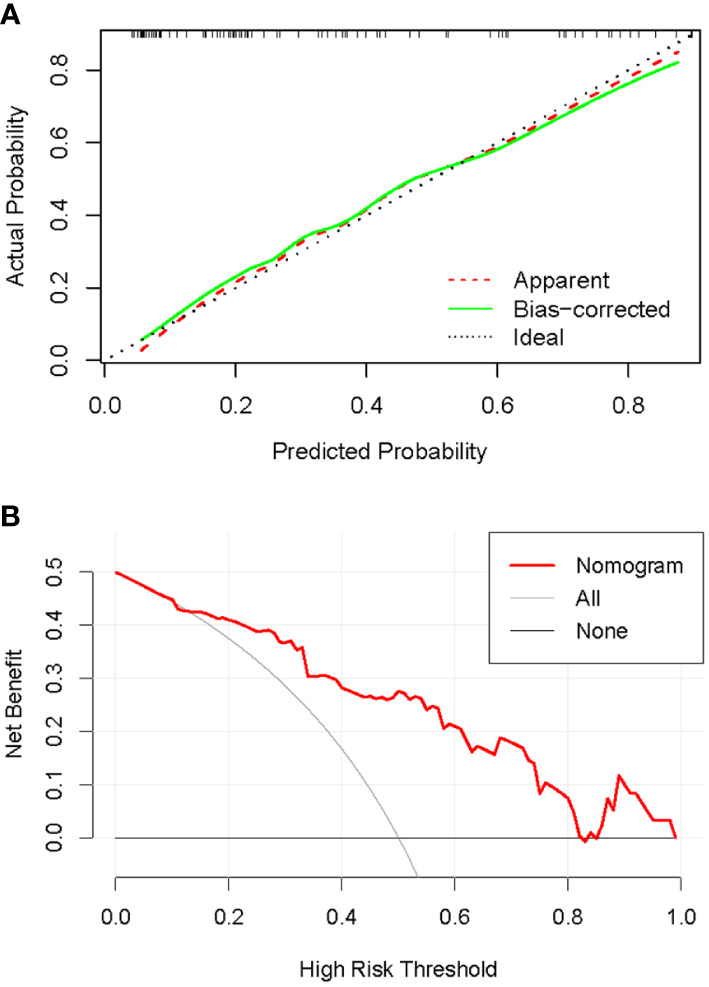
Calibration and decision curves of the nomogram. **(A)**: Calibration curve of the nomogram. The diagonal line represented the ideal performance, the red dashed line represented the actual performance, and the solid green line represented the corrected performance. There was a good agreement between predicted performance and actual performance. **(B)**: Decision curve of the nomogram. The nomogram achieved the maximum benefit compared to all treatments (gray curve) and all no treatments (black horizontal line).

The C-index of the nomogram was 0.837, which indicates high performance. Moreover, the calibration curves showed good consistency between predictability and actual status (**Figure 9A**). Finally, we used decision curve analysis to determine whether the nomogram would help implement clinical treatment strategies. The decision curve showed that within the threshold probability range of 0.2 to 0.8, the nomogram had the maximum net benefit compared to the “all treatment” strategy and the “all no treatment” strategy (**Figure 9B**), which indicated high clinical applicability.

## Discussion

In this retrospective study, we innovatively developed a nomogram based on MRI radiomics and semantic features to predict the +7/-10 status in IDH-wt histologically LGG for the first time, providing a convenient and quantitative prediction tool for clinicians. We developed six radiomics models and determined the optimal based on their performance in the independent test group. The nomogram demonstrated superior performance in both the training and test groups compared to the LDA+DC model and clinical model. The combination of semantic and radiomics features was more effective in predicting +7/-10 in IDH-wt histologically LGG than either radiomics or semantic features alone, indicating that both macroscopic and microscopic information from medical imaging were indispensable in revealing tumor heterogeneity (25, 26). Moreover, the calibration curve and c-index of the nomogram confirmed its acceptable predictive performance and stability. The decision curve analysis further proved the clinical utility of the nomogram.

It is widely recognized that tumor heterogeneity is closely related to the biological behavior and prognosis of tumors. Chromosomal number aberration is the most common genomic abnormality in human tumors, and the specific aneuploidy often leads to the activation of oncogenes or loss of suppressor genes (3). Chr7 gain and Chr10 loss, the two most common genomic alterations in gliomas, are essential events in molecular glioblastoma development and are very important prognostic biomarkers (27). Yadav et al. proposed that the loss of Chr10 leads to the inactivation of tumor suppressor genes annexin A7 and the phosphatase and tensin homolog (PTEN), promoting the oncogenic epidermal growth factor signaling pathway and increasing tumorigenesis (10). Senhaji et al. found that the loss of Chr10 leads to the methylation of methylguanine methyltransferase (MGMT) at 10q26, which is an important way to increase the sensitivity of tumor cells to alkylating agents. Therefore, adjuvant chemotherapy with temozolomide or nitrosourea is the best choice for LGG with Chr10 loss, especially for the elderly who have difficulty receiving radiotherapy and chemotherapy simultaneously (3). In addition, the gain of the entire Chr7 and loss of heterozygosity at Chr10 are associated with shorter survival time in adult patients with LGG (9). Therefore, accurate identification of +7/-10 helps determine prognostic stratification and personalized treatment of IDH-wt histologically LGG.

In clinical practice, stereotactic biopsy is the basic method for the diagnosis of brain tumors. However, it is an invasive examination and suffers from unsatisfactory reproducibility and inter-observer agreement. Conventional MRI examination, as a widely available imaging method, has become the best tool for the initial diagnosis of tumors and long-term postoperative monitoring due to its excellent tissue resolution and non-radiation. Radiomics extracts microscopic features from image data using computers and transforms them into quantifiable data, establishing a connection between medical imaging and genomics (28). In addition, radiomics allows the analysis of intact tumors and overcomes the dilemma of insufficient samples for molecular and histopathological examination. Nowadays, models based on MRI radiomics features have been proven to accurately predict the molecular expression status of gliomas. Zheng et al. effectively predicted the expression grade of the synaptophysin gene in LGG using multi-parametric MRI radiomics (29). Casale et al. developed a machine learning model based on MRI radiomics features to predict chromosome 1p/19q co-deletion in LGG, and achieved an accuracy of 0.72 in the independent test cohort (14).

In our study, we used 2 feature selection methods and 3 machine learning algorithms to compare the performance of different radiomics models. In the independent test group, the LDA+DC model achieved the best performance with an AUC of 0.736 and an accuracy of 0.750. LDA aims to separate two classes by searching for a linear combination of predictive variables that maximizes the separation between groups. As a robust linear classification algorithm, LDA has shown excellent performance in some previous radiomics-related studies. In the study of Sied et al., LDA can effectively predict the postoperative pseudo-progression in patients with glioblastoma, with an AUC value of 0.93 (30). Tian et al. developed an LDA model based on radiomics features to distinguish glioblastoma from anaplastic astrocytoma. The accuracy of the model in the validation cohort was 0.968, demonstrating the promising potential for clinical applications (31). It should be noted that in our study, the models based on the NN algorithm performed well in the training group but did not achieve satisfactory performance in the test group. We believe that this over-fitting phenomenon may be mainly attributed to insufficient training samples. NN is an algorithm that simulates the structure of human neurons and can perform complex logical operations and reveal the nonlinear relationship between input variables and output results. NN is capable of adaptively adjusting the weights of nodes in the model to optimize the performance and thus has been widely used in various scenarios (32, 33). However, NN is weak in processing linear data and prone to over-fitting when the training data is insufficient (34). Therefore, selecting the appropriate machine learning algorithm is a crucial factor to ensure the model performance.

Additionally, we found that in the training group, the median AUC values of the models based on DC features were higher than that based on LASSO in 10-fold cross-validation. In the test group, except for the NN algorithm, the AUC values of the models based on DC features were higher than that of the corresponding models based on LASSO. This indicated that the feature selection method played a pivotal role in improving the performance of the models. Among the 10 features selected by DC, there were 9 filtering features, which may be due to the fact that filtering features could better display the local information of the images. Filtering can adjust the frequency and time windows according to the image grayscale and signal characteristics, which is beneficial for enhancing image details (35). However, filtering methods smooth or sharpen the images, which inevitably weakens the interpretability and biological connotation of the features while improving their stability. This is an urgent issue that needs to be addressed in current radiomics-related research.

In the radiomics signature selected by DC, there existed an original feature termed “Original_GLCM_Correlation_T2”, which was a texture feature of the gray-level co-occurrence matrix (GLCM) subgroup in the T2WI sequence. Correlation is a value between 0 (uncorrelated) and 1 (perfectly correlated) that characterizes the correlation between the grayscale values and the respective voxels in the GLCM (36). This feature has previously been described as a robust and independent feature in other clinical scenarios (37, 38). A larger correlation value indicates a more uniform gray level variation within the ROI. In this study, the IDH-wt histologically LGG with +7/-10 exhibited a lower median correlation value in the T2WI sequence compared to those without +7/-10, indicating a more uneven grayscale distribution at the microscopic level, which may be due to the higher frequency of necrosis. In our study, the frequency of necrosis in IDH-wt histologically LGG patients with +7/-10 was significantly higher in both the training group (*P*=0.015) and test group (*P*=0.030) than in patients without +7/-10. This indicates that conventional radiological features and microscopic radiomics features are in harmony with each other. Zhang et al. developed a nomogram based on MRI semantic features to predict DNA copy number subtypes in LGG (39). They found a significant association (*P* < 0.05) between tumor necrosis and the CN2 subtype, which was indicative of a poorer outcome (the shortest overall survival among CN1, CN2, and CN3). Tian et al. developed a nomogram to predict the TERT promoter expression status in high-grade gliomas, and the study showed that tumor necrosis was closely related to TERT promoter mutations (40). The value of semantic features in revealing the heterogeneity and biological phenotype of tumors cannot be ignored, and further studies are needed to confirm it.

Our study still has some limitations. Firstly, this single-center study may be subject to regional and selection biases, which may result in the included patients not being fully representative of the epidemiological distribution. Therefore, multi-center studies involving heterogeneous populations are necessary in the future. Moreover, further validation in external cohorts also helps to confirm the generalization ability of the nomogram we developed. Secondly, there are a large number of proven machine learning algorithms available, but they may be suitable for different scenarios. In our research, we also found that appropriate algorithms are the key to achieving good model performance. Therefore, more models need to be developed in the future to improve the classification performance. Finally, the specific biological significance of the radiomics features still needs further exploration in the future.

## Conclusion

In summary, the nomogram based on radiomics and clinical semantic features can predict the +7/-10 status of IDH-wt histologically LGG patients non-invasively, which may help with risk stratification and individualized treatment planning for glioma patients, and also aid in the diagnosis of molecular glioblastoma.

## Data availability statement

The raw data supporting the conclusions of this article will be made available by the authors, without undue reservation.

## Ethics statement

The studies involving humans were approved by the institutional review board of Beijing Tiantan Hospital. The studies were conducted in accordance with the local legislation and institutional requirements. Written informed consent for participation was not required from the participants or the participants’ legal guardians/next of kin in accordance with the national legislation and institutional requirements.

## Author contributions

JM and XK: conception and design. YM, XK, FX, YL, and YQL: collection and assembly of data. XK and YM: data analysis and interpretation. XK and YM: article writing. All authors contributed to the article and approved the submitted version.
