# Supplementary material for: Development of a nomogram based on radiomics and semantic features for predicting chromosome 7 gain/chromosome 10 loss in IDH wild-type histologically low-grade gliomas
Source: Front Oncol. 2023 Sep 15;13:1196614. doi: 10.3389/fonc.2023.1196614 (PMC10541227; doi:10.3389/fonc.2023.1196614)
Supplement: Supplementary file 1 [file DataSheet_1.docx]

**Supplementary material**

**S1. Image acquisition parameters**

The acquisition parameters of T1WI sequence were: repetition time (TR), 1900-2400ms, echo time (TE), 2.95-19.8ms, slice thickness, 5mm, matrix, 512×512. The acquisition parameters of T2WI sequence were: TR, 3100-4500ms, TE, 99-114.21ms, slice thickness, 5mm, matrix, 512×512. The acquisition parameters of FLAIR sequence were: TR, 4800-9000ms, TE, 130-256.34ms, slice thickness, 5mm, matrix, 512×512. The acquisition parameters of CET1 were as follows: TR, 1900-2000ms, TE: 8.6-20ms, slice thickness, 5mm, matrix, 512×512. In the above four sequences, the slice gap was 1 mm. Gd-diethylenetriamine pentaacetic acid (dose:0.1 mmol/kg) was used as the contrast agent for contrast enhancement imaging.

**S2. Introduction of radiomics features**

The radiomics features in our research were extracted using the Pyradiomics library (http://pyradiomics.readthedocs.io/). The bin width used for feature extraction is 25. In this study, four groups of features were extracted for each patient, including: (1) first-order features (n=18); (2) shape features (n=14); (3) texture features (n=75); (4) filtering features (n=1488). First-order features directly describe the distribution of voxel intensities within the image region defined by the mask through commonly used and basic metrics. The shape feature includes descriptors of the 2D and 3D size and shape of the ROI. Texture features are commonly used to describe the spatial arrangement of image intensity. The groups of texture features in our research included Gray Level Co-occurrence Matrix (GLCM, n=24), Gray Level Size Zone Matrix (GLSZM, n=16), Gray Level Run Length Matrix (GLRLM, n=16), Neighbouring Gray Tone Difference Matrix (NGTDM, n=5) and Gray Level Dependence Matrix (GLDM, n=14). Filtering features are a set of features obtained by mathematical transformations based on first-order or texture features. Seven filtering methods including wavelet, Laplacian of Gaussian (LoG), square, square-root, exponential, logarithm and gradient were applied in our research. The wavelet transform decomposes the first-order features and texture features in the original image into low-frequency and high-frequency in three dimensions respectively, and each first-order feature and texture feature will be decomposed into 8 wavelet features. Each first-order and texture feature will be smoothed and sharpened using LoG filters with kernel sizes of 1mm, 2mm, and 3mm, respectively. In addition, all first-order features and texture features will be filtered by square, square root, exponential, logarithm and gradient operations respectively to obtain the corresponding filtering features. After 7 kinds of filtering transformation, we got a total of 1488 (744+279+93+93+93+93+93) filtering features. Finally, we obtained 1595 (14+18+75+1488) radiomics features for each sequence. Since we analyzed 4 sequences, each patient finally extracted a total of 6380 features.

**S3. Description of the** **semantic characteristics**

We selected 12 common clinical factors and imaging findings including age, gender, location, side, margin, contrast enhancement, necrosis, multifocal, restricted diffusion, deep white matter invasion, hemorrhage, and ependymal involvement as potential clinical risk characteristics. The radiological features were evaluated by two neuro-radiologists with more than 5 years of experience who were blinded to the clinical and pathological information of the patients. In case of disagreement, a senior neuro-radiologist with 10 years of experience would re-evaluate the features. Tumor shapes were defined as regular or oval, and irregular or lobulated. The location of the lesion was defined as frontal and non-frontal lobes. The side of the lesion center was defined as the right, left, and bilateral, respectively. In terms of the margin, if most of the outside non-enhancing boundary of the lesion is well-defined and smooth, it will be defined as a distinct margin; whereas if the boundary is unclear and irregular, it will be defined as an indistinct margin. Contrast enhancement was defined as all or part of the tumor showing a recognizable higher signal on the post-comparison T1WI sequence compared to the pre-contrast T1WI sequence. Necrosis was defined as an irregular region within the tumor that did not enhance or show markedly diminished enhancement, was hyperintense on T2W images and hypointense on T1W images. Multifocal was defined as having at least one region of the tumor, either enhancing or non-enhancing, which was not contiguous with the dominant lesion and was outside the region of signal abnormality (edema) surrounding the dominant mass. Restricted diffusion referred to regions with hyperintense on DWI sequences and corresponding low apparent diffusion coefficient (ADC) values. Deep white matter invasion referred to lesions extending into the internal capsule, corpus callosum, or brainstem. Hemorrhage referred to any intrinsic foci of hypointense on T2WI or hyperintense on T1WI. Ependymal involvement referred to any invasion of the adjacent ependymal surface in continuity with enhancing or non-enhancing tumor matrix.

**S4. Optimal radiomics signatures**

In feature selection, distance correlation (DC) selected the top 10 most important features by calculating the distance correlation coefficient. Based on the 1-SE criterion, the least absolute shrinkage and selection operator (LASSO) determined the optimal value of λ to be 0.1039153 through 10-fold cross-validation and selected 3 features. The features selected by the two algorithms were shown in **Supplementary Table 1**.

**Supplementary Table 1** The radiomics signatures selected by the two algorithms

| Algorithm | Feature name |
| --- | --- |
| DC | ① log.sigma.1.0.mm.3D_glszm_SmallAreaEmphasis_CET1;  ② exponential_gldm_DependenceEntropy_T2;  ③ wavelet.HHL_firstorder_Mean_T1;  ④ square_glszm_LowGrayLevelZoneEmphasis_T1;  ⑤ original_glcm_Correlation_T2;  ⑥wavelet.LHH_ngtdm_Busyness_T2;  ⑦ wavelet.HLH_glszm_LargeAreaLowGrayLevelEmphasis_T2;  ⑧ exponential_firstorder_Uniformity_T2;  ⑨ exponential_glrlm_LongRunHighGrayLevelEmphasis_T2;  ⑩ gradient_glszm_SmallAreaLowGrayLevelEmphasis_T2 |
| LASSO | ① wavelet.HHL_glcm_Imc1_T2  ② wavelet.LHH_ngtdm_Busyness_T2  ③ wavelet.HLH_glszm_LargeAreaLowGrayLevelEmphasis_T2 |
